# Real-World Efficacy of Intravitreal Faricimab for Diabetic Macular Edema: A Systematic Review

**DOI:** 10.3390/jpm14090913

**Published:** 2024-08-28

**Authors:** Safiullah Nasimi, Nasratullah Nasimi, Jakob Grauslund, Anna Stage Vergmann, Yousif Subhi

**Affiliations:** 1 Department of Ophthalmology, Odense University Hospital, 5000 Odense, Denmark; safiullah_nasimi@hotmail.com (S.N.); nasimi_11@hotmail.com (N.N.); jakob.grauslund@rsyd.dk (J.G.); anna.stage.vergmann@rsyd.dk (A.S.V.); 2 Department of Clinical Research, University of Southern Denmark, 5230 Odense, Denmark; 3 Steno Diabetes Center Odense, Odense University Hospital, 5000 Odense, Denmark; 4 Department of Ophthalmology, Vestfold Hospital Trust, 3103 Tønsberg, Norway; 5 Department of Ophthalmology, Rigshospitalet, 2100 Copenhagen, Denmark; 6 Department of Clinical Medicine, University of Copenhagen, 1172 Copenhagen, Denmark

**Keywords:** diabetic macular edema, faricimab, anti-VEGF, real-world, efficacy, systematic review

## Abstract

Background: Diabetic macular edema (DME) is a prevalent exudative maculopathy, and anti-vascular endothelial growth factor (anti-VEGF) therapy is the first-line choice for treatment. Faricimab, a novel anti-VEGF and anti-angiopoietin-2 bispecific agent, has recently been approved for the treatment of DME. In this study, we systematically reviewed the real-world evidence of the efficacy of faricimab for the treatment of DME. Methods: We searched 11 databases for eligible studies. Study selection and data extraction were made independently by two authors in duplicate. Eligible studies were reviewed qualitatively. Results: We identified 10 eligible studies that summarized data from a total of 6054 eyes with a mean follow-up of between 55 days and 12 months. Five studies reported outcomes in a population of both treatment-naïve and previously treated eyes, and five studies reported outcomes exclusively in relation to eyes that were previously treated. Faricimab improved the best-corrected visual acuity and macular thickness. The extension of the treatment interval was possible in 61–81% of treatment-naïve eyes and 36–78% of previously treated eyes. Conclusions: Faricimab for DME yields clinical outcomes similar to those known from previous anti-VEGF treatments but with extended treatment intervals, thus lowering the burden of therapy for patients. Long-term real-world studies are warranted.

## 1. Introduction

Diabetic macular edema (DME) is a prevalent exudative maculopathy and the primary cause of visual impairment in patients with diabetes [1,2,3]. The pathophysiology of DME involves the disruption of the blood–retina barrier through damage to the retinal capillary endothelium and the pericytes [4]. Leakage through the disrupted blood–retina barrier leads to macular edema [4]. These changes are orchestrated by a range of pro-inflammatory signaling molecules, which include vascular endothelial growth factor (VEGF) [4]. Since the introduction of anti-VEGF therapy for DME, the prognosis for the patients has improved dramatically, and anti-VEGF therapy has become the mainstay of DME treatment [5].

Although studies show that in DME, the principal burden of therapy with anti-VEGF is in the first years [3,5], many patients experience a heavy burden of treatment with injections and visits every 4–6 weeks. This has to be accommodated in addition to visits to other health providers due to the co-morbidities of diabetes and routine care of diabetes [3,6,7].

Recently, faricimab (Vabysmo^®^, Roche AG, Basel, Switzerland) was approved by the United States Food and Drug Administration and the European Medicines Agency for the treatment of DME. Faricimab is a novel anti-VEGF and anti-angiopoietin-2 (Ang-2) bispecific agent, which, apart from the VEGF, also addresses the pro-inflammatory Ang-2 and its role in promoting vascular permeability [8]. The agency approval was based on the two phase 3 trials, YOSEMITE and RHINE, which compared aflibercept in a fixed-dose regimen every 8 weeks to faricimab in a fixed-dose regimen every 8 weeks and faricimab in a treat-and-extend regimen [8]. Both studies demonstrated that faricimab in a treat-and-extend regimen was superior to aflibercept in a fixed regimen [8]. In addition, faricimab therapy was able to provide superiority with a proportion of eyes in extended treatment intervals [8]. Thus, faricimab holds promise for lowering the burden of therapy while achieving satisfactory outcomes in eyes with DME. However, when generalizing the results from clinical trials to real-world contexts, the results may deviate [9,10]. Real-world patients may differ from those that fit the strict eligibility criteria of clinical trials, and routine clinical organization and routine clinical practice may differ from the circumstances in a controlled trial [9,10]. Thus, to better understand the real-world efficacy of faricimab for DME, we systematically reviewed the available literature.

## 2. Materials and Methods

### 2.1. Study Protocol and Registration

We followed the recommendations of the Cochrane Handbook for the design and conduct of our study [11]. Our protocol is registered at PROSPERO (protocol no. CRD42024537105). We followed the Preferred Reporting Items for Systematic Reviews and Meta-Analysis (PRISMA) [12]. According to Danish law, no institutional review board approval is relevant for systematic reviews.

### 2.2. Eligibility Criteria

We defined eligible studies for this review as those that fulfilled the following eligibility criteria:

*Population:* Studies of patients with DME. We did not restrict the patient population based on any previous treatment. We only considered studies of human patients.

*Exposure:* Intravitreal injection therapy using faricimab 6 mg (0.05 mL). There were no restrictions with respect to the number of injections for patients included.

*Outcomes:* Change from baseline to follow-up in BCVA and macular thickness, as well as the burden of therapy (i.e., the number of injections/therapies needed). We did not restrict the study to any definition of macular thickness and accepted the study authors’ definitions (i.e., central retinal thickness, CRT; central macular thickness, CMT; central subfield thickness, CSFT).

*Study design:* Any prospective or retrospective studies with original data of real-world evidence were included. We also included case reports and non-peer-reviewed publications and conference abstracts but only considered studies disseminated in English for practical purposes. This strategy was employed as the field is relatively new, and any delay to publication may restrict available evidence for review. No restriction was made on the geographical origin of the study or the date of study publication.

### 2.3. Information Sources, Literature Search, and Study Selection

One trained author (Y.S.) conducted a systematic literature search in 11 databases (PubMed, Embase, Web of Science Core Collection, BIOSIS Previews, Current Contents Connect, Data Citation Index, Derwent Innovations Index, KCI-Korean Journal Database, ProQuest Dissertations & Theses Citation Index, SciELO Citation Index, and the Cochrane Library). All searches were conducted on 12 April 2024. The literature search details for individual databases are available in Supplementary File S1.

One author (Y.S.) removed all duplicates and obviously irrelevant reports. Two authors (S.N. and N.N.) independently screened the full text of the remaining records for eligible studies. Reference lists were screened for further eligible studies. Disagreements between authors were discussed until consensus, and if consensus could not be reached, a third author (Y.S.) made the final decision.

### 2.4. Data Collection, Data Extraction, Risk of Bias within Studies, and Data Synthesis

Two authors (S.N. and N.N.) extracted the data and evaluated the risk of bias within studies. Data on the study and population characteristics, treatment details, and clinical outcomes at baseline and follow-up were extracted. Since we expected studies to be primarily retrospective cohort studies, we used the Newcastle-Ottawa Scale for the evaluation of the risk of bias within studies [13]. Disagreements between authors were discussed until consensus, and if consensus could not be reached, a third author (Y.S.) would make the final decision. All studies were reviewed in text and in tables. Meaningful meta-analyses were not possible due to heterogeneity in populations, treatment regimens, and follow-ups across studies.

## 3. Results

### 3.1. Study Selection Process

**Figure 1:**
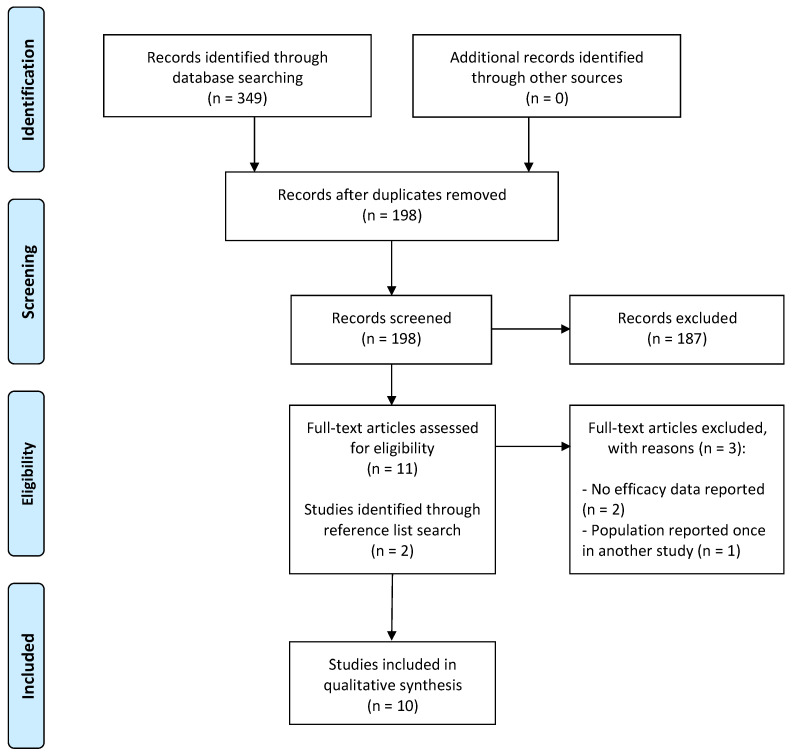
PRISMA flow diagram of the study selection process. Number (n) of studies in each category is highlighted throughout the study selection process.

Our literature search identified a total of 349 records. Of these, 151 were duplicates, and 187 were obviously irrelevant records. All remaining 11 records were retrieved and examined as full-text studies. Three of these studies (after examining their full text) did not fulfill our eligibility criteria. We reviewed reference lists thoroughly and identified two additional eligible studies. Thus, 10 studies were identified as being eligible for our systematic review. The study selection process is outlined in Figure 1.

### 3.2. Study and Population Characteristics 

The 10 eligible reports of real-world evidence comprised six full-text original papers and six conference abstracts [14,15,16,17,18,19,20,21,22,23]. Treatment regimens investigated across studies were subject to a large variation; some studies reported outcomes from a single injection, several consecutive injections, or as-needed therapy in a longer follow-up period. The studies followed the patients for a mean period that ranged from 55 days to 12 months. Details of the study characteristics are outlined in Table 1. 

The studies summarized data from a total of 6054 eyes. These were based on 5167 patients, although this number does not include the number of patients from one study with 12 eyes, which did not report the number of patients. One report was a multinational study, whereas the remaining studies reported data from patients in the USA (4 studies), Japan (3 studies), and the UK (2 studies). The mean age ranged between 62 and 69 years. Biological males comprised between 39 and 79% of the population in individual studies. Five studies reported outcomes in a population of both treatment-naïve and previously treated eyes, five studies reported outcomes exclusively from eyes that were previously treated, and no study reported exclusively on treatment-naïve eyes. Details of the population characteristics are summarized in Table 2.

The baseline characteristics of the studied eyes are summarized in Table 3.

### 3.3. Results of Individual Studies

The work of Bailey et al. is a EURETINA conference abstract from the FARWIDE-DME study, which was a retrospective study in the United Kingdom based on data extraction from electronic medical records from multiple hospital sites [14]. This study included both treatment-naïve eyes and previously treated eyes (the majority being treated with aflibercept). However, the reasons for the therapy switch were not reported [14]. After the third injection, the BCVA increased with 6.0 ETDRS letters (−0.12 decrease in logMAR) in treatment-naïve eyes and 1.4 ETDRS letters (−0.03 decrease in logMAR) in previously treated eyes [14]. These improvements in BCVA were stated as being statistically significant, but further details regarding the statistics were not reported [14]. After the 5th injection, 81% of treatment-naïve eyes and 52% of the previously treated eyes were extended to ≥8-week intervals [14].

Durrani et al. reported experiences of switching to faricimab therapy when treating eyes with persistent DME despite anti-VEGF therapy (the majority were treated with aflibercept) [15]. Before switching to faricimab, 51% were treated every 4 weeks and the rest between every 5–8 weeks [15]. After the switch to faricimab and a mean follow-up of 3.2 ± 1.1 months, the injection interval was extended by ≥2 weeks in 36% of cases [15]. The BCVA improved from 0.40 ± 0.30 logMAR to 0.38 ± 0.27, but this was not statistically significant (*p* = 0.4) [15]. The mean CFT decreased from 380 ± 155 µm before the switch to 324 ± 147 after the switch (*p* < 0.001) [15].

The work of Ibrahim et al. is an ARVO conference abstract of a single-center experience with a faricimab loading dose for DME in both treatment-naïve eyes and previously treated eyes [16]. Details on previous treatments and the reason for the therapy switch were not reported. The CMT was 460 µm at the baseline, and this decreased at a mean value of −124 µm [16]. This study neither reported BCVA data nor evaluated the statistical significance of the changes [16].

Kusuhara et al. reported experiences from a single center on faricimab therapy for DME [17]. This study included both treatment-naïve eyes and previously treated eyes (the majority being treatment with aflibercept); however, the reason for the therapy switch was not reported [17]. Eyes were treated in a pro re nata regimen, and therefore, interval extension as a phenomenon was not reported [17]. In 24% of the eyes, there was a history of vitrectomy, and these eyes received significantly more faricimab injections than the non-vitrectomized eyes (0.6 injections/month vs. 0.3 injections/month, *p* = 0.04) [17]. From the baseline to 6 months, the BCVA remained unchanged, whereas the CRT decreased from 401 ± 97 µm to 328 ± 99, although this change did not have statistical significance (*p* = 0.07) [17]. In 29% of eyes, the treating physician decided to switch from faricimab to another anti-VEGF, such as triamcinolone or vitrectomy, due to insufficient treatment effects [17].

Ohara et al. reported experiences with switching to faricimab therapy in eyes with persistent DME despite aflibercept or ranibizumab therapy [18]. The decision to switch to faricimab was based on inadequate resolution or an increase in DME despite aflibercept or ranibizumab therapy within 8 weeks [18]. Eyes were treated in a pro re nata regimen, and therefore, the interval extension as a phenomenon was not reported [18]. After the switch, the recurrence of DME was observed at a mean of 10.8 ± 4.9 weeks, and 78% of the patients had a longer recurrence interval compared to their previous anti-VEGF therapy [18]. The BCVA remained unchanged from the baseline and throughout the follow-ups, whereas the CMT decreased from 473 ± 222 µm to 327 ± 153 µm, although this change did not have statistical significance (*p* = 0.09) [18]. A reduction of 20% or more in the CME was achieved at 4 months in 56% of cases and at the final visit in 67% of cases [18].

Patel et al. presented an ARVO conference abstract about a single-center experience with faricimab in two consecutive doses for DME in eyes previously treated with other anti-VEGF [19]. Details on previous treatments and the reasons for the therapy switch were not reported [19]. The BCVA improved with −0.05 ± 0.27 logMAR, and the CRT improved with −33 ± 48 µm, although these improvements did not reach statistical significance [19].

Rush et al. reported a 1-year experience of switching from aflibercept to faricimab for DME within a single center [20]. The cases for inclusion were those identified as being aflibercept-resistant, which was defined as receiving ≥6 aflibercept treatments during the preceding 12 months and ≥4 aflibercept treatment during the preceding 6 months [20]. Upon switching to faricimab, the patients’ eyes received a loading dose with three injections, which was then followed by a treat-and-extend regimen [20]. After 1 year, an extension to a ≥8-week treatment interval was achieved in 39% of cases [20]. From the baseline to 12 months, the BCVA improved from 0.60 logMAR to 0.47 logMAR (*p* < 0.01), and the CMT decreased from 400 µm to 340 µm (*p* < 0.01) [20].

The work of Sheth et al. is a conference poster for the 56th Retina Society Annual Scientific Meeting, presenting the 3-month data from the prospective real-world observational multinational multicenter study VOYAGER [21]. In the study, there was a subset of eyes with DME, apart from two treatment-naïve eyes (2% of the population); the DME study population was comprised of eyes previously treated with anti-VEGF (any anti-VEGF, including faricimab, which comprised 66% of those previously treated) [21]. From the baseline to 3 months, the CST decreased from 311 µm to 269 µm, and the BCVA improved from 70 ETDRS letters to 72 ETDRS letters, although this study did not report the statistical significance of these changes [21].

The work of Tabano et al. is an ARVO conference abstract of the FARETINA-DME study, which utilized the American IRIS registry [22]. We also identified an ASRS conference presentation from the same study with further information [24]. This study included both treatment-naïve eyes and previously treated eyes (the majority being treated with aflibercept); however, the reason for the therapy switch was not reported [22,24]. After the fourth injection, the BCVA remained largely unchanged in both treatment-naïve eyes and previously treated eyes [22]. An extension of the fourth injection was possible in 61% of the treatment-naïve eyes and 64% of the eyes previously treated with another anti-VEGF [22,24].

Takamura et al. reported on macular changes after a loading dose of faricimab in eyes previously treated with anti-VEGF for DME [23]. Neither the reason for the therapy switch nor details on previous anti-VEGF therapy were reported [23]. From the baseline to 12 weeks, BCVA improved, and CRT decreased at a statistically significant level (*p* = 0.04 and *p* < 0.0001, respectively), although the exact values were only reported in figures and were not extractable from the manuscript text [23]. This study focused on the microaneurysm turnover and found a total microaneurysm reduction of 41% after the commencement of faricimab therapy [23].

### 3.4. Risk of Bias within the Studies

The Newcastle–Ottawa Quality Assessment Scale for Cohort Studies, which we used for risk of bias within the studies in this review, included two items that dealt with “selection of the non-exposed cohort” and “comparability of cohorts” (within individual studies), which were irrelevant for our topic of interest. Therefore, for these items, we stated “irrelevant” (annotated with —), as we decided that it would be a more accurate response. One item dealt with whether the outcome of interest was present at the start of the study, which was a difficult topic for a study of anti-VEGF efficacy on DME, partly because of the fluctuating nature of the macular edema and partly because many studies evaluated the effect of switching therapy from another agent, which presumably had a certain level of efficacy. Therefore, for this item, we stated “unclear” (annotated with /), as we decided that it would be a more accurate response. The other items, which dealt with participant representativeness, ascertainment of exposure, assessment of outcome, follow-up length, and adequacy, all fulfilled the scale criteria for one star for each item. Thus, all studies in review achieved a total quality score of 5 (Table 4).

## 4. Discussion

In this systematic review of 10 studies covering 6054 eyes, we find that faricimab has been applied in different real-world settings in various treatment regimens. Overall, faricimab is reported to have a satisfactory efficacy, at least in terms of expectations for anti-VEGF therapy for DME in general. When used in a treat-and-extend regimen, an important proportion of individuals could be treated with long treatment intervals. In eyes previously treated with other anti-VEGF therapies, faricimab was able to provide a similar level of efficacy with a lower burden of therapy. These findings are in line with those reported in the YOSEMITE and RHINE phase 3 trials [8]. After 1 year, the faricimab treat-and-extend group was able to extend treatment intervals to every 12 weeks in 20.1–21.0% of cases and to every 16 weeks in 51.0–52.8% of cases [8]. Although the efficacy of faricimab in terms of any improvement in the BCVA and the CRT seems comparable to that of aflibercept, both in the real-world studies outlined in this systematic review and those reported in the YOSEMITE and RHINE phase 3 trials, there is an important value in being able to provide these outcomes while also reducing the burden of therapy for the patients. The 2-year efficacy results of the YOSEMITE and RHINE trials were recently published [25]. The faricimab treat-and-extend group was able to extend treatment intervals to every 12 weeks in 13.6–18.1% of cases and to every 16 weeks in 60.0–64.5% of cases at week 96 [25]; in other words, fewer patients in the 12-week group and more patients in the 16-week group. Future real-world studies will determine if these results are also reproduced in real-world settings.

Faricimab is also approved for the treatment of neovascular age-related macular degeneration (AMD) [26]. Real-world efficacy studies of faricimab for neovascular AMD also show a pattern of BCVA improvement and CRT decrease similar to that of aflibercept but with a longer duration [27,28,29]. Considering that exudative maculopathies are expected to increase in the future with an aging demographic as a driving force [3,30,31,32], solutions are needed to accommodate other challenges in real-world settings that are often not considered in controlled trials, e.g., capacity issues, staffing, and the burden of visiting a treatment center monthly/bimonthly for years. In that regard, from a clinical practice/real-world setting point of view, treatments with a longer duration, such as that provided by faricimab, provide a welcome change.

In healthy retinae, Ang-2 is expressed at very low levels in the deep vascular plexus [33,34]. However, hypoxia and hyperglycemia in DME can induce a pro-inflammatory retinal milieu that promotes the expression of Ang-2 [33,34]. In the presence of other pro-inflammatory signal molecules, the activity of Ang-2 is enhanced, which also leads to pericyte loss and the weakening of endothelial cell junctions [33,34]. Ang-2 amplifies the response of VEGF as retinal vessels become more sensitive to VEGF through Ang-2 activity [33,34]. Experimental studies show that the absence of Ang-2 attenuates cytokine-induced leukocyte adhesion and VEGF-induced vascular leakage [33,34]. These preclinical findings support the pathophysiological role of Ang-2 in DME and provide a rationale for a longer duration of therapy.

The limitations of our systematic review should be acknowledged. First, the current evidence base is limited to five full-text studies and five conference abstracts. Only one study provided insight into outcomes beyond 6 months, and the longest follow-up was 1 year. Real-world studies of the long-term efficacy of faricimab for DME are needed to understand outcomes beyond the first year. Second, five of the studies are conference abstracts, which are not peer-reviewed. Although the inclusion of grey literature is recommended by the Cochrane Handbook [11], the interpretation of such studies should be made with great care as they are not peer-reviewed. Third, apart from a few studies, the rationale for switching to faricimab remained unclear. There is an important difference between switching due to the lack of effect of the previous anti-VEGF therapies and switching when anti-VEGF is an efficacious treatment option, but there is a need for a longer treatment interval. Finally, as demonstrated in the RETAIN study [35], DME treat-and-extend and pro re nata regimens provide similar outcomes in terms of BCVA and CSFT. However, the treat-and-extend group received more injections, whereas the pro re nata group had more clinical controls [35]. Based on the local costs and practicalities related to the number of visits and injections, some centers may find it more desirable to provide DME treatment using a pro re nata approach [3]. Currently, no controlled trial or long-term real-world evidence exists on the efficacy of faricimab vs. other anti-VEGF therapies when faricimab is used in a pro re nata approach.

## 5. Conclusions

Studies on the real-world efficacy of faricimab for DME show similar outcomes as previous anti-VEGF in terms of BCVA and macular thickness but with extended treatment intervals and, thus, a lower burden of therapy for the patients. Long-term studies are needed to evaluate long-term efficacy in real-world settings.

## Figures and Tables

**Table 1:** Study characteristics.

Reference	Study Design	Treatment and Follow-Up Regimen	Duration of Follow-Up
Bailey et al., 2023 [14]	Retrospective cohort study	≥1 faricimab injections in routine clinical practice without further definition of the treatment regimen.	3.9 ± 2.9 months
Durrani et al., 2024 [15]	Retrospective cohort study	≥3 consecutive faricimab injections and ≥1 clinical visit with complete data. Details of the interval between the injections or the clinical visits were not reported; however, the short follow-up period indicates a follow-up after the loading dose.	3.2 ± 1.1 months
Ibrahim et al., 2023 [16]	Retrospective cohort study	Details of the number of injections or clinical visits were not reported.	3 months
Kusuhara et al., 2023 [17]	Retrospective cohort study	Patients were followed monthly in a pro re nata regimen.	5.5 ± 2.0 months
Ohara et al., 2023 [18]	Retrospective cohort study	Patients were followed with an individualized follow-up interval in a pro re nata regimen.	6.1 ± 1.3 months
Patel et al., 2023 [19]	Retrospective cohort study	Two consecutive faricimab injections. Details of the interval between the injections or the clinical visits were not reported.	Unclear
Rush et al., 2023 [20]	Retrospective cohort study	Patients received monthly faricimab injections for 3 months, after which treatments and follow-ups were based on a treat-and-extend regimen.	12 months
Sheth et al., 2023 [21]	Prospective cohort study	Routine clinical practice without further definition of the treatment regimen, number of injections, or clinical visits.	3 months
Tabano et al., 2023 [22]	Retrospective cohort study	Registry-based study of individuals with ≥4 faricimab injections. Details of the treatment regimens were not available. An extended interval was defined as those cases in which injections were performed >6 weeks apart.	55.2 ± 48.0 days
Takamura et al., 2024 [23]	Prospective cohort study	Patients received monthly faricimab injections for 3 months. Follow-ups were made monthly until 1 month after the final injection.	3 months

**Table 2:** Population characteristics.

Reference	Country	Age, Mean ± SD	Biological Males, %	Patients, N
Bailey et al., 2023 [14]	UK	63 ± 12 years	61%	1921
Durrani et al., 2024 [15]	USA	69 ± 9 years	67%	53
Ibrahim et al., 2023 [16]	USA	N/A	39%	46
Kusuhara et al., 2023 [17]	Japan	68 ± 7 years	79%	19
Ohara et al., 2023 [18]	Japan	69 ± 10 years	39%	18
Patel et al., 2023 [19]	UK	62 ± 20 years	N/A	N/A
Rush et al., 2023 [20]	USA	62 ± 10 years	49%	51
Sheth et al., 2023 [21]	Multinational	65 ± 12 years	61%	69
Tabano et al., 2023 [22]	USA	68 ± 10 years	54%	2962
Takamura et al., 2024 [23]	Japan	68 ± 7 years	61%	28

Abbreviations: N = number; N/A = not available; SD = standard deviation; UK = United Kingdom; USA = United States of America.

**Table 3:** Baseline characteristics of study eyes.

Reference	Treatment-Naïve Eyes, N	Previously Treated Eyes, N	Best-Corrected Visual Acuity	Central Retinal Thickness
Bailey et al., 2023 [14]	952	1721	For treatment-naïve eyes and previously treated eyes, respectively, no data in 5.7% and 6.4%, ≤55 ETDRS letters in 26.2% and 22.4%, and ≥56 ETDRS letters in 68.1% and 71.3%	N/A
Durrani et al., 2024 [15]	0	69	0.40 ± 0.30 logMAR	380 ± 155 µm
Ibrahim et al., 2023 [16]	9	11	N/A	Mean 460 µm
Kusuhara et al., 2023 [17]	14	7	0.24 ± 0.24 logMAR	401 ± 97 µm
Ohara et al., 2023 [18]	0	18	0.23 ± 0.28 logMAR	474 ± 222 µm
Patel et al., 2023 [19]	0	12	N/A	N/A
Rush et al., 2023 [20]	0	51	Mean 0.60 logMAR	Mean 400 µm
Sheth et al., 2023 [21]	105	2	Mean 70 ETDRS letters	Mean 311 µm
Tabano et al., 2023 [22]	415	2615	≥20/40 Snellen in 45% of treatment-naïve eyes and 48% of previously treated eyes	N/A
Takamura et al., 2024 [23]	0	53	Mean 0.32 logMAR	Mean 432 µm

Abbreviations: ETDRS = early treatment of diabetic retinopathy study; N = number; N/A = not available; logMAR = logarithm of the minimal angle of resolution. BCVA and CRT are summarized as mean ± standard deviation unless such data were not available. In such cases, we summarized the best available data and reported the summary statistics used.

**Table 4:** Risk of bias within the individual studies included in the review.

	Selection				Comparability	Outcome			Quality Score
Reference	#1	#2	#3	#4	#1	#1	#2	#3	
Bailey et al., 2023 [14]	✯	—	✯	/	—	✯	✯	✯	5
Durrani et al., 2024 [15]	✯	—	✯	/	—	✯	✯	✯	5
Ibrahim et al., 2023 [16]	✯	—	✯	/	—	✯	✯	✯	5
Kusuhara et al., 2023 [17]	✯	—	✯	/	—	✯	✯	✯	5
Ohara et al., 2023 [18]	✯	—	✯	/	—	✯	✯	✯	5
Patel et al., 2023 [19]	✯	—	✯	/	—	✯	✯	✯	5
Rush et al., 2023 [20]	✯	—	✯	/	—	✯	✯	✯	5
Sheth et al., 2023 [21]	✯	—	✯	/	—	✯	✯	✯	5
Tabano et al., 2023 [22]	✯	—	✯	/	—	✯	✯	✯	5
Takamura et al., 2024 [23]	✯	—	✯	/	—	✯	✯	✯	5

The Newcastle–Ottawa Quality Assessment Scale for Cohort Studies evaluates categories within three domains: Selection, Comparability, and Outcome. The categories within Selection are (#1) representativeness of the exposed cohort, (#2) selection of the non-exposed cohort, (#3) ascertainment of exposure, and (#4) demonstration that the outcome of interest was not present at the start of the study. For Comparability, only one category was evaluated in (#1) comparability of cohorts based on the design or analysis. The categories within Outcome are (#1) assessment of outcome, (#2) was follow-up long enough for outcomes to occur, and (#3) adequacy of the follow-up of cohorts. The quality score is a summary of the number of stars across all categories within each study.

## Data Availability

No new data were created or analyzed in this study. Data sharing is not applicable to this article.

## Supplementary Materials

The following supporting information can be downloaded at https://www.mdpi.com/article/10.3390/jpm14090913/s1, Supplementary File S1: Documentation of the literature search.
