# Supplementary material for: Real-World Efficacy of Intravitreal Faricimab for Diabetic Macular Edema: A Systematic Review
Source: J Pers Med. 2024 Aug 28;14(9):913. doi: 10.3390/jpm14090913 (PMC11432955; doi:10.3390/jpm14090913)

Supplementary File S1. Documentation of the literature search.

PubMed:

History and Search Details — Download — Delete

Search	Actions	Details	Query	Results	Time
#1	•••	⌄	Search: **faricimab AND ("diabetic macular edema" OR "diabetic macular oedema" OR "diabetic maculopathy")** Sort by: **Most Recent**<br>("faricimab"[Supplementary Concept] OR "faricimab"[All Fields]) AND ("diabetic macular edema"[All Fields] OR "diabetic macular oedema"[All Fields] OR "diabetic maculopathy"[All Fields])<br>**Translations**<br>**faricimab:** "faricimab"[Supplementary Concept] OR "faricimab"[All Fields]	51	14:34:42

Showing 1 to 1 of 1 entries

Embase:

```
Embase <1974 to 2024 April 12>

1         faricimab.mp. [mp=title, abstract, heading word, drug
trade name, original title, device manufacturer, drug
manufacturer, device trade name, keyword heading word, floating
subheading word, candidate term word]         397
2         faricimab/        385
3         "diabetic macular edema".mp. [mp=title, abstract, heading
word, drug trade name, original title, device manufacturer, drug
manufacturer, device trade name, keyword heading word, floating
subheading word, candidate term word]         10806
4         "diabetic macular oedema".mp. [mp=title, abstract,
heading word, drug trade name, original title, device
manufacturer, drug manufacturer, device trade name, keyword
heading word, floating subheading word, candidate term word]
          1358
5         "diabetic maculopathy".mp. [mp=title, abstract, heading
word, drug trade name, original title, device manufacturer, drug
manufacturer, device trade name, keyword heading word, floating
subheading word, candidate term word]         792
6         diabetic macular edema/    9104
7         1 or 2   397
8         3 or 4 or 5 or 6  11645
9         7 and 8  170
```

Cochrane Central:

Search Name:
Date Run:15/04/2024 02:45:42
Comment:

ID	Search	Hits
#1	faricimab89	
#2	"diabetic macular edema"	2006
#3	"diabetic macular oedema"	2006
#4	"diabetic maculopathy"	78
#5	#1 AND (#2 OR #3 OR #4)	40

Web of Science Core Collection, BIOSIS Previews, Current Contents Connect, Data Citation Index, Derwent Innovations Index, KCI-Korean Journal Database, ProQuest Dissertations & Theses Citation Index, and SciELO Citation Index:

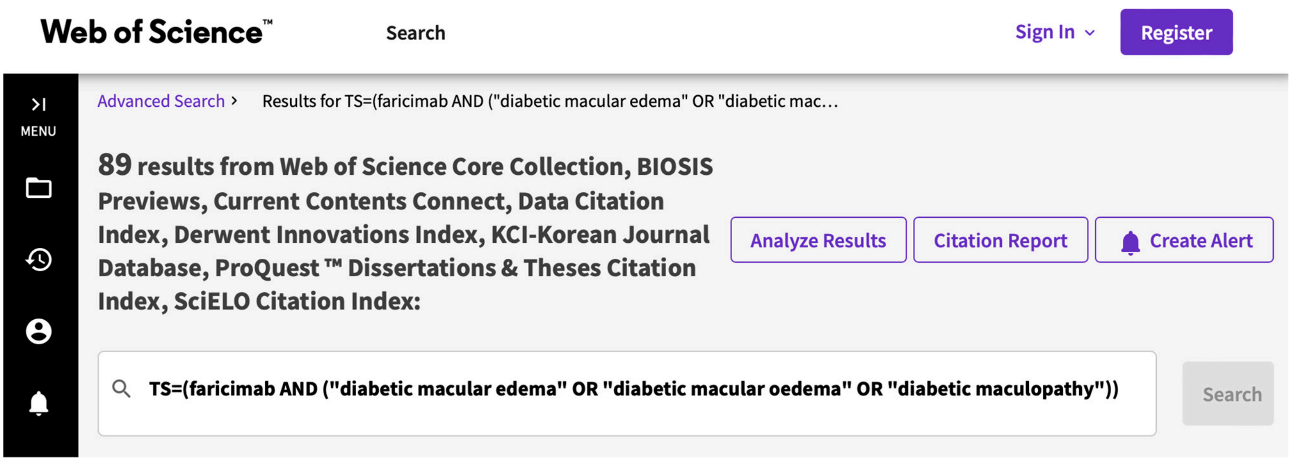

Supplement: Supplementary file 1 [file jpm-14-00913-s001.zip › jpm-3153659-Supplementary File S1.pdf]
